# Invasive Physiological Assessment: From Binary to Continuous

**DOI:** 10.36660/abc.20200054

**Published:** 2020-02

**Authors:** Daniel Chamié, Alexandre Abizaid

**Affiliations:** 1 Invasive Cardiology Department, Instituto Dante Pazzanese de Cardiololgia, São Paulo, SP - Brazil; 2 Invasive Cardiology Department, Hospital do Coração, São Paulo, SP - Brazil; 3 Invasive Cardiology Department, Hospital Sírio Libanês, São Paulo, SP - Brazil

**Keywords:** Myocardial Ischemia, Fractional Flow Reserve, Myocardial Coronary Artery Disease, Coronary Stenosis, Risk Factors, Percutaneous Coronary Intervention

Described by Pijls et al., in 1993, and based on extensive validation and robust clinical data, fractional flow reserve (FFR) was incorporated into the guidelines of myocardial revascularization to guide the need for revascularization of angiographically intermediate stenosis in patients with stable coronary artery disease (CAD).^1-3^ The broadest arguments for this decision were: (1) by depicting a complex tridimensional structure as a planar silhouette coronary angiography suffers from well-known limitations, it presents large variability in estimating coronary stenosis severity, and it has low ability in predicting the functional significance of epicardial coronary stenoses, and (2) revascularization in stable coronary artery disease based solely on the severity of luminal narrowing, as determined by coronary angiography, does not improve clinical outcomes as compared to optimized medical treatment^8^ or versus revascularization of only physiologically significant lesions.^9-11^

The central premise of invasive assessment of coronary physiology is to identify myocardial ischemia with superior spatial resolution (per vessel) compared to non-invasive methods (per territory), aiding in the identification of lesions (and, therefore, patients) that are more likely to benefit from revascularization. However, despite the clinical benefits and guideline recommendations, the FFR uptake in clinical practice remains low (< 10%) in most catheterization laboratories around the globe. Costs, time added to procedures, patient discomfort to hyperemic stimulus or contraindications to adenosine use, as well as difficulties in interpretation of physiological traces in certain anatomic situations (e.g., serial/diffuse stenosis), are some of the reasons for FFR underutilization.

Recently, the introduction of instantaneous wave-free ratio (iFR) led to renewed interest in the use of invasive physiology. The iFR is measured at rest - without the need to achieve maximal hyperemia -, which simplifies the use of coronary physiology in several anatomic scenarios, with shorter procedure time and fewer adverse symptoms for the patient. Seven years after its initial description by Sen et al.,^12^ two large randomized studies documented the non-inferiority of iFR compared with FFR on the occurrence of adverse clinical outcomes when they were used to guide revascularization of coronary stenoses.^13,14^ These results were achieved despite a classification mismatch between FFR and iFR in approximately 20% of the cases.^15^

In this issue of the *Arquivos Brasileiros de Cardiologia*, Vieira *et al*^16^ describe their initial experience with the use of iFR to guide coronary revascularization decision-making in 96 lesions from 52 patients, accumulated for over four years. Out of these, 56 stenoses (58.3%) were graded as intermediate (between 41% and 70%), and 40 (41.7%) were classified as severe (between 71% and 90%), as determined by visual assessment of coronary angiography. In agreement with extensive previous validation, the authors used a cut-off value of iFR of ≤ 0.89^15^ to classify stenoses as hemodynamically significant and decide upon the need for revascularization. Percutaneous coronary intervention (PCI) with stent implantation was the primary outcome used, which was performed in 32% of all studied lesions. However, the median and the interquartile range of iFR observed in intermediate (0.92 [0.82 to 0.94]) and severe (0.79 [0.61 to 1.00]) lesions draw our attention to the fact that a non-negligible proportion of lesions were treated with stent despite the absence of physiological significance as per the iFR evaluations - particularly those of intermediate severity (Figure 4, Vieira et al.^16^). These findings corroborate the idea that physiological information is just one (important) piece of the decision-making puzzle, which should take into account other equally important factors, such as clinical presentation, presence, type and frequency of anginal symptoms, target lesion location, left ventricular function and perspective of long-term prognosis.

Although relieving significant stenosis through mechanical intervention improves anginal symptoms more effectively than optimal medical treatment,^17,18^ this practice does not result in major significant reductions of hard clinical events such as death and myocardial infarction.^8^ It is noteworthy that about half the patients with a positive FFR have a favorable long-term prognosis when maintained on optimal medical therapy alone.^19,20^ Thus, there is a significant opportunity for medical optimization of some stable patients regardless of the physiological significance of the lesion under investigation, particularly in asymptomatic or oligosymptomatic individuals with lesions that produce minimal physiological impact. These arguments leave room for disagreements with the outcome adopted by Vieira et al,^16^ which was the performance of PCI or not. On the contrary, a much more complex and thorough assessment (including the physiological evaluation) should support the revascularization decision than simply the “positive” or “negative” value of a diagnostic index.

Although the clinical decision for revascularizing coronary stenoses is binary, which ends up justifying the search for cut-off points that determine the choice of one strategy over another, we advocate that invasive coronary physiology should be assessed in a more comprehensive, continuous and interpretative manner. In this sense, similarly to what was demonstrated in the classical study by Hachamovitch et al.,^21^ robust evidence indicates a linear association between FFR and the risk of adverse cardiac outcomes. Adverse outcome rates increased proportionally with reduced FFR values, revealing a risk continuum, far beyond a fixed cut-off point.^22,23^ In addition, lesions with lower FFR values are the ones which receive the greatest absolute benefits from PCI.^23^ On the other hand, for lesions with FFR values around the cut-off point, the benefits of revascularization are lower and at times uncertain.

Although ischemia determined at the vessel level - in other words, “positive” or “negative”, as the sum of all lesions throughout the artery length - has been the traditional basis for FFR utilization, a series of technological advances have allowed for a more global and systematic approach to assessing the presence of myocardial ischemia. Through manual pullback of the pressure sensor, the non-hyperemic iFR index allows for the assessment of the functional impact of each lesion along the target vessel segment. Moreover, overlaying these results onto the angiographic images provides a valuable functional-anatomical co-registration. This technique yields a more accurate characterization on the distribution of the physiological effects of coronary heart disease, enabling the diagnosis of focal and diffuse disease (which frequently coexist in the same vessel), in addition to quantifying the contribution of each for the iFR value at the artery level. Furthermore, it is possible to simulate several PCI strategies and estimate the physiological results of the possible intervention. Hence, the result is an evolution from the binary negative/positive to a more comprehensive assessment of the physiological impact of CAD, and the potential benefits of PCI, in case this is the chosen therapeutic strategy. This concept proved to be particularly important in the recent DEFINE-PCI^24^ pilot study. In a population of 500 patients undergoing PCI with stent implantation, whose procedures were considered successful by angiographic criteria, iFR pullback showed that 24% of the patients treated remained with physiologically significant stenoses. It is worth mentioning the finding that in more than 80% of the cases, the abnormal iFR matched focal stenoses, which are easily treatable, reaffirming the limitations of angiography in identifying coronary flow-limiting lesions. In cases with serial lesions or diffuse disease, the hyperemic flow through one stenosis is affected by the presence of another stenosis in the same artery, making interpretation of FFR values challenging in this frequent anatomic subset. On the other hand, resting flow is stable across almost the entire range of epicardial coronary stenosis severity. Thus, changes in resting pressure are more predictable, and the contribution of each stenosis along the vessel can be more easily estimated, representing a practical advantage of iFR over FFR.^25,26^

Therefore, we believe that the introduction of new indexes (e.g. angiography-derived FFR, coronary computed tomography-derived FFR, resting indexes, among others) and new possibilities of understanding the functional effects of coronary stenosis have promoted growing interest in invasive and non-invasive assessment of cardiac physiology in the “post-FFR era”. We keep waiting the development of new physiological tools that enable the measurement of myocardial ischemia in an easier and more accurate way (instead of using surrogate outcomes), as well as tools to simplify the study of the coronary microcirculation status. These advances will contribute to a more individualized approach to coronary revascularization decision-making, better understanding of focal and diffuse disease, and treatment of post-MI patients whose microcirculation has been impaired. Until then, we advance our application of physiological assessment, from binary to continuous.
